# Fluoride-Ion-Responsive Sol–Gel Transition in an L-Cysteine/AgNO_3_ System: Self-Assembly Peculiarities and Anticancer Activity

**DOI:** 10.3390/gels10050332

**Published:** 2024-05-14

**Authors:** Dmitry V. Vishnevetskii, Yana V. Andrianova, Elizaveta E. Polyakova, Alexandra I. Ivanova, Arif R. Mekhtiev

**Affiliations:** 1 Department of Physical Chemistry, Tver State University, Building 33, Zhelyabova Str., Tver 170100, Russia; nuri-chan-87@mail.ru (Y.V.A.); elizabeth03pol@gmail.com (E.E.P.); 2 Institute of Biomedical Chemistry, 10 Building 8, Pogodinskaya Str., Moscow 191121, Russia; 3 Department of Applied Physics, Tver State University, Building 33, Zhelyabova Str., Tver 170100, Russia; alex.ivanova33@yandex.ru

**Keywords:** cysteine–silver sol, fluoride anions, supramolecular hydrogels, self-assembly, microstructure, squamous cell carcinoma, cytotoxicity

## Abstract

Supramolecular hydrogels based on low-molecular-weight compounds are a unique class of so-called “soft” materials, formed by weak non-covalent interactions between precursors at their millimolar concentrations. Due to the variety of structures that can be formed using different low-molecular-weight gelators, they are widely used in various fields of technology and medicine. In this study, we report for the first time an unusual self-assembly process of mixing a hydrosol obtained from L-cysteine and silver nitrate (cysteine–silver sol—CSS) with sodium halides. Modern instrumental techniques such as viscosimetry, UV spectroscopy, dynamic light scattering, zeta potential measurements, SEM and EDS identified that adding fluoride anions to CSS is able to form stable hydrogels of a thixotropic nature, while Cl^−^, Br^−^ and I^−^ lead to precipitation. The self-assembly process proceeds using a narrow concentration range of F^−^. An increase in the fluoride anion content in the system leads to a change in the gel network morphology from elongated structures to spherical ones. This fact is reflected in a decrease in the gel viscosity and a number of gel–sol–gel transition cycles. The mechanism of F^−^’s interaction with hydrosol includes the condensation of anions on the positive surface of the CSS nanoparticles, their binding via electrostatic forces and the formation of a resulting gel carcass. In vitro analysis showed that the hydrogels suppressed human squamous carcinoma cells at a micromolar sample concentration. The obtained soft gels could have potential applications against cutaneous malignancy and as carriers for fluoride anion and other bioactive substance delivery.

## 1. Introduction

Molecular self-assembly is one of the most common processes in nature. It is believed that it plays an essential role in the formation, sustenance and evolution of life cycles [1]. The main scientific research in this field focus mainly on the study of biomacromolecules: proteins [2], nucleic acids [3] and polysaccharides [4]. Herewith, less attention is paid to low-molecular-weight compounds. The self-assembly process in such systems, for example, in an aqueous medium, leads to the unusual phenomenon of gel formation, which is observed at a low content of the dispersed phase [5]. Such gels are supramolecular objects. Supramolecular hydrogels formed from low-molecular-weight gelators (LMWGs) have a number of advantages. In addition to the amphiphilicity of the molecules and the participation of non-covalent interactions (hydrogen bonding, π-π stacking, Coulomb forces, etc.) in gel formation (as in biological structures), they are characterized by affordability, high purity, a well-known chemical structure, the biocompatibility of their initial components and ease of gelation [6].

The use of amino acids as LMWGs provides a unique opportunity for the construction of supramolecular hydrogels that cannot be obtained on the basis of traditional organic/inorganic molecules. Some of the first who showed the possibility of gelation in such systems were teams of authors who studied the process of self-assembly in aqueous and aqueous organic media, where molecules of substituted amino acid derivatives acted as the dispersed phase: N-lauroyl-L-alanine [7] and N-stearoyl-L-glutamic acid [8]. A little later, the ability of supramolecular gels to form based on L-glycine and L-alanine [9] and other amino acids was demonstrated [10,11,12,13,14]. Even then, the potential of using these gels in various fields of technology and medicine was shown.

Spatial network formation in LMWG-based supramolecular gels is caused by weak intermolecular forces, which are responsible for the reversible sol–gel transition. Thus, they can respond to external stimuli: temperature, pH or ions [15,16,17,18,19]. Stimuli-responsive supramolecular gels have attracted the most attention in various fields such as drug delivery [20,21,22], biosensors [23,24], environment remediation [25,26], molecular photoswitches [27] and self-healing materials [28,29,30]. The use of different anions as initiators of the gel formation of LMWGs [31] is of particular interest because of their essential role in the environment and many biological processes [32,33,34]. Our scientific group found that a system based on aqueous solutions of L-cysteine and various silver salts is capable of forming a stable hydrosol [35,36,37,38,39,40] at quite a low dispersed phase concentration (0.01%). The self-assembly process is caused by the high affinity of silver ions to the thiol group of the amino acid. Furthermore, sol is prone to the formation of a supramolecular gel when initiated using low-molecular-weight anions, e.g., salts of various metals. The effect of double-charged anions such as sulfate, molybdate and tungstate on the gelation process has been studied in detail [35]. However, the role of single-charge and those most important in the human body, such as halide anions, has not been discovered. Due to the fact that sols obtained on the basis of sulfur-containing amino acids and silver salts have shown various properties, such as anticancer [36,37], photocatalytic [38], antibacterial/antibiofilm [39] and film-forming [40] properties, the need to investigate the influence of various biologically active compounds on hydrosol colloidal stability and activity is relevant.

The present work deals with investigation of the peculiarities of supramolecular gel formation based on cysteine–silver sol (CSS) and halide anions. Iodide, bromide and chloride ions led to the precipitation of CSS nanoparticles. Herewith, it has been shown for the first time that fluoride anions can initiate the sol–gel transition process in the system under study. The obtained cysteine–silver gels (CSGs) possess thixotropic properties. The viscosity of the gels drops as the anion concentration increases, which is related to changes in the gel network microstructure from elongated motifs to spherical particles. The interaction of the CSS particles with F^−^ has an electrostatic nature. In vitro experiments demonstrated that CSGs have a high toxicity to cancer SiHa cells and a moderately toxic effect on normal Wi-38 ones. Thus, novel stimuli-responsive supramolecular hydrogels, along with their unusual self-assembly process of formation, could rouse academic and application interest in the future.

## 2. Results and Discussion

### 2.1. Visual Analysis of Sol–Gel Transition Process

**Figure 1:**
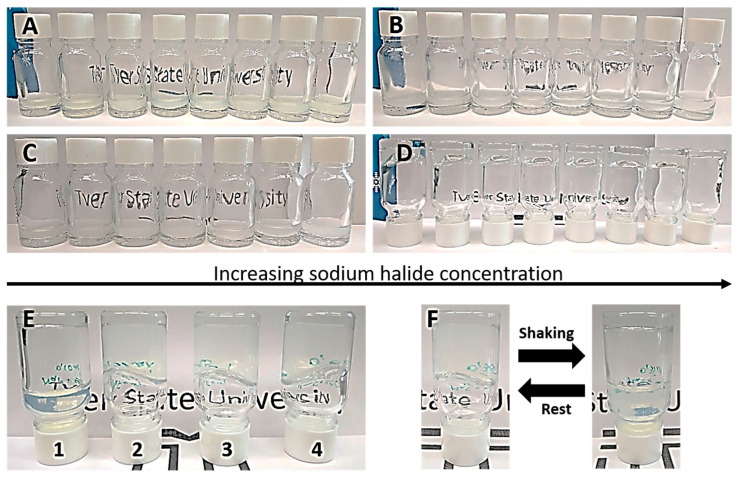
The CSS’s behavior after addition of halide ions: (**A**) iodide, (**B**) bromide, (**C**) chloride, (**D**) fluoride in the concentration range of 0.2–2.6 mM for (**A**–**C**) and 2.8–4.4 mM for (**D**). (**E**) F^−^-based-CSG at anion concentrations (mM) of **1**—0.9, **2**—3.1, **3**—3.5, **4**—3.9. (**F**) Thixotropic properties of F^−^-based CSG.

At the beginning of our research, we explored the behavior of CSS with the addition of halide ions and a sequential increase in their concentration (Figure 1). One can see the formation of yellow or white/yellow precipitates of varying intensities for the systems with I^−^ and Br^−^ (Figure 1A,B). Hydrogel formation was observed in terms of the interaction of Cl^−^ and F^−^ with the hydrosol. Herewith, the Cl^—^-based CSGs were destroyed after 1–7 days of resting in a dark place with the formation of white precipitates (Figure 1C). The gels based on F^−^ were characterized by high stability in their native state for more than 6 months, and no opalescence was detected (Figure 1D,E). Furthermore, these CSGs possessed thixotropic properties (Figure 1F). According to our early investigations, the interaction of L-cysteine with silver ions leads to the medium’s acidification (pH = 2.6) and the formation of nanoparticles constructed from a silver nanoparticle (AgNP) “core” and L-cysteine/Ag^+^ complex inner “shell” [35,39]. The outer side of the “shell” is formed by amino and carboxyl groups. The isoelectric point (pI) of such a system is 5.5 [41], that is, the nanoparticles have a positive surface charge value, which is responsible for the sol’s colloidal stability. The system behaves like globular protein. Thus, the halide ions tend towards strong binding with the surface of the CSS particles. Differences in hydrogel formation are probably related to two factors. The first one is the ability of the ions to structure water molecules. In accordance with the lyotropic series of Hofmeister [42], the kosmotropic properties of halide anions decrease in the following order: F^−^ > Cl^−^ > Br^−^ > I^−^. Therefore, fluoride ions have the highest structuring ability. The Hofmeister theory is suitable for our systems since it was made based on the interactions of proteins with various salts. The second one is the specific interactions of halide ions with the surface of the CSS particles, mostly with Ag^+^ incorporated into the shell. It is well known that the solubility equilibrium (SE) changes as 1.7 × 10^−10^–5 × 10^−13^–1 × 10^−16^ at 25 °C for silver chloride, bromide and iodide, respectively. Thus, they are not soluble in water, except for AgF. Furthermore, the nucleophilicity grows going down the periodic table (F^−^ < Cl^−^ < Br^−^ < I^−^) for polar protic solvents [43], that is, F^−^ is the softest anion in its interaction with the surface of the CSS nanoparticles. Literature analysis shows [5,31] the various LMWGs undergo only gel–sol transitions under the action of F^−^. Thereby, we have demonstrated for the first time that fluoride anions can lead to a reversible sol–gel transition, whose peculiarities have been studied in the present paper using modern instrumental techniques.

### 2.2. Characterization of Gels Based on Fluoride Anions

**Figure 2:**
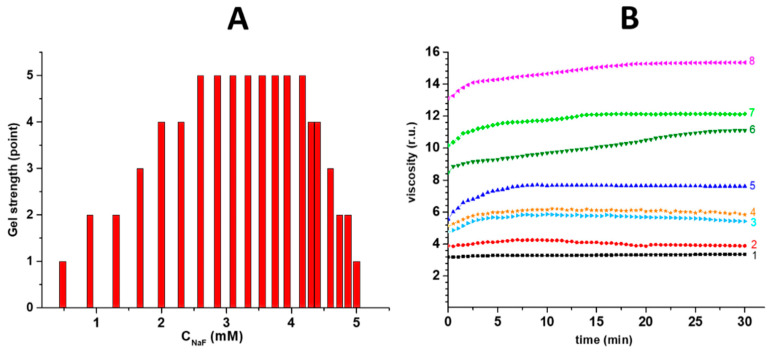
(**A**) The concentration diagram for F^−^-based CSG. (**B**) The viscosity dependence on time for F^−^-based CSGs at various concentrations of anions (mM): **1**—0.0, **2**—0.9, **3**—1.7, **4**—4.6, **5**—2.6, **6**—3.9, **7**—3.5, **8**—3.1.

The interaction of CSS particles with fluoride anions leads to the formation of stable hydrogels in a rather narrow concentration range (Figure 2A). The gel strength was visually evaluated after turning the test tube over by 180 degrees (Figure 1F). A corresponding integer from 0 to 5 was assigned to a given state [35]. The gel strength’s dependence on the anion concentration is dome-shaped. The visual data were fully confirmed according to the results of vibrational viscosimetry (Figure 2B); however, for the gels with a gel strength point of 5, the higher the concentration of the fluoride ions, the lower the viscosity. Due to the fact that the gel network is formed from the initial sol nanoparticles, it is necessary to consider the features of self-assembly at the micro- and nano-levels.

**Figure 3:**
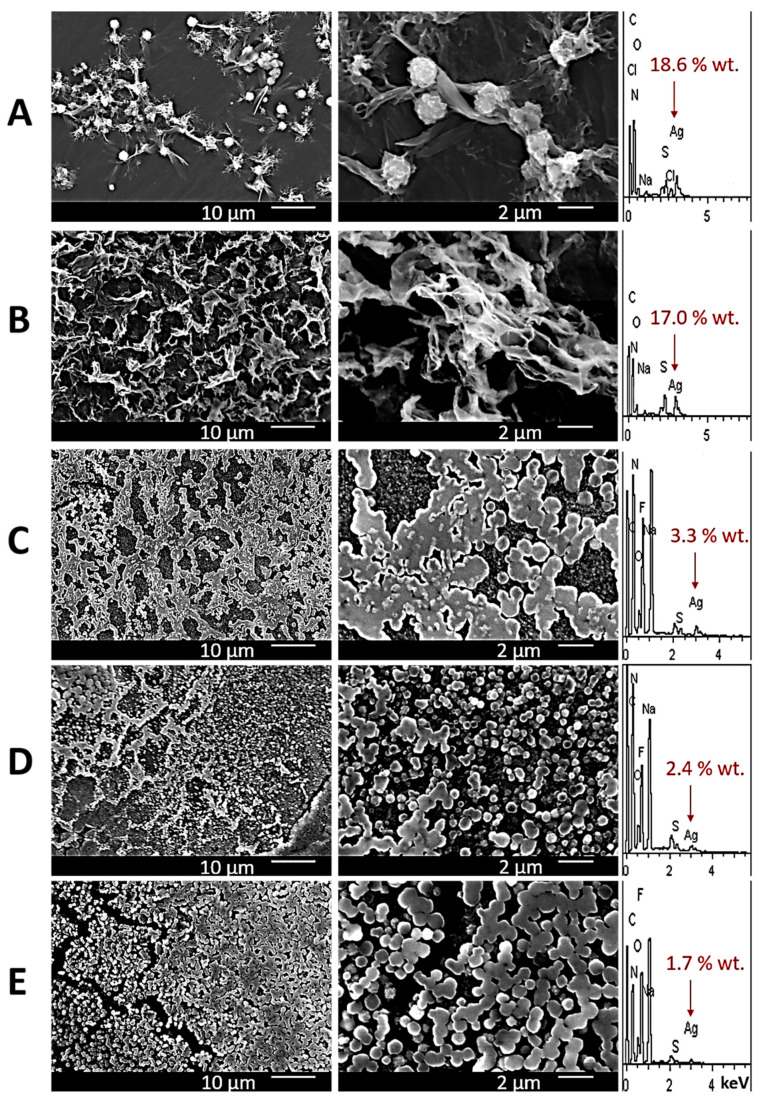
SEM images and EDS of CSGs based on (**A**)—chloride; (**B**) sulfate; (**C**–**E**) fluoride anions at anion concentrations (mM) of 3.1, 3.5 and 3.9, respectively.

The mechanical properties of gels are related to the peculiarities of the gel network microstructure. Indeed, increasing the fluoride ion concentration leads to morphological changes in the gel carcass (Figure 3C–E), the elements of which reduce their surface area by moving from more elongated structures to spherical ones with an elementary grain diameter from 0.1 to 2 µm. These results explain the data obtained using viscosimetry. It should be noted that after several cycles of mechanical destruction of the gels, only those in which the content of fluoride ions was minimal could be restored. Hydrogels based on Cl^−^ and SO_4_^2−^ anions are presented as systems for comparison (Figure 3A,B): the gel based on the former system is destroyed quickly. As was mentioned above, the gel network has a loose structure built from single spherical aggregates; the latter gel system is a well-studied one, and it shows quite a dense structure consisting of intersecting ribbon-like fibers. The results of the elemental analysis verified the SEM data (Figure 3): on moving from using a SO_4_^2−^-based gel to using F^−^-based ones and increasing the fluoride concentration, the Ag content on the surface of the gel network decreases.

**Figure 4:**
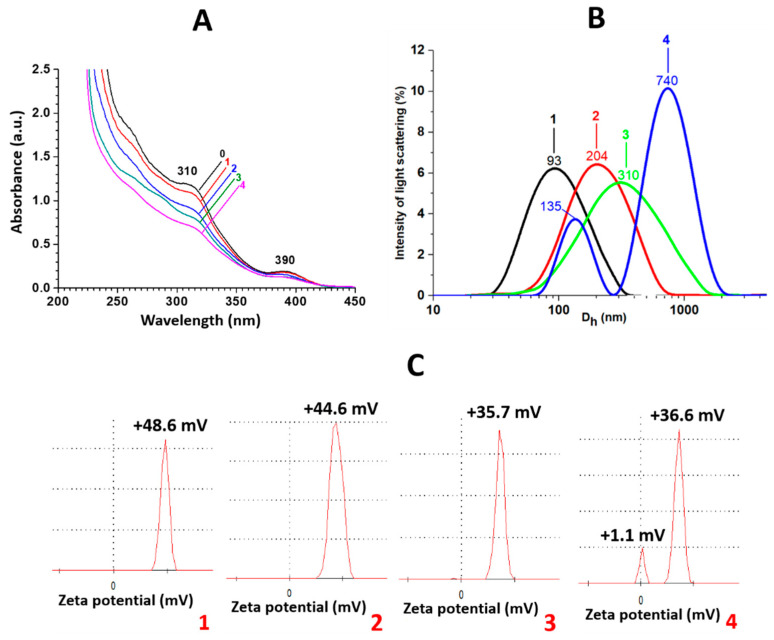
(**A**) UV spectra, (**B**) particle size distribution and (**C**) zeta potential measurements for F^−^-based CSGs at different concentrations of anions (mM): **0**—0.0, **1**—0.9, **2**—2.6, **3**—3.1, **4**—3.9.

The UV spectra of the gels are presented in Figure 4A. One can see that the addition of the fluoride anions to the CSS does not change the position of the absorption bands at 310 and 390 nm, corresponding to argentophilic interactions in the L-cysteine/Ag^+^ complexes and the surface plasmon resonance of the crystalline phase of the silver nanoparticles [39]. This suggests that fluoride ions do not influence the electronic structure of the CSS nanoparticles but interact only with their surface. A similar behavior was observed for the sulfate anions [35,37]. The dynamic light scattering data indicate a unimodal particle size distribution (Figure 4B). An increase in the content of the fluoride ions in the system leads to growth in the particle size and a drop in the polydispersity coefficient, while the distribution becomes bimodal. The hydrodynamic diameter of the CSS particles is 50 nm [39]. Measurements of the zeta potentials of the systems are given in Figure 4C. One can see a consistent decrease in the values of the zeta potential with an increase in the concentration of the fluoride anions. Furthermore, an increase in the anion content in the system is reflected in the appearance of a second peak at the zero value of the zeta potential. The zeta potential for CSS is +60 mV [35,39]. This value is mainly determined by fully protonated amino groups according to the pI of the system [36,37,39,44].

### 2.3. Proposed Mechanism of Gel Formation

**Figure 5:**
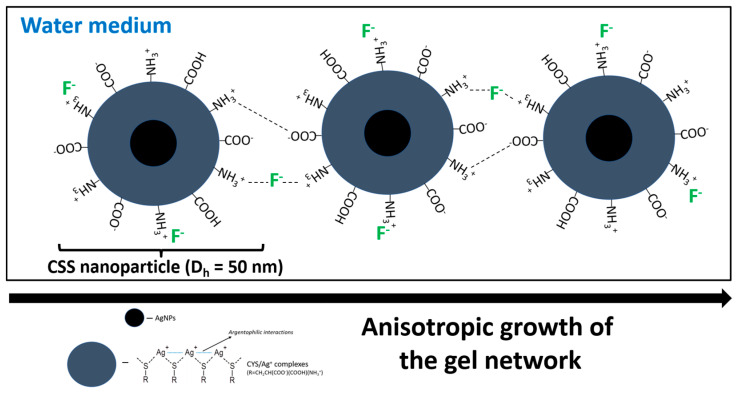
The proposed mechanism of self-assembly in CSS after addition of F^−^.

Summing up the above data, one can suggest the following stage-by-stage mechanism of self-assembly (Figure 5):

(1) In accordance with the zeta potential measurements, particle size distribution and UV analysis, the fluoride ions condense on the positively charged surface of the CSS nanoparticles and cause the suppression of their charge, which leads to a decrease in the distance between the particles and their interactions, with the formation of larger aggregates; the data from the literature indicate that the F^−^ acts as a destructive agent in the process of gelation for various LMWGs regardless of the solvent polarity [31]. Herewith, the process of gel-to-sol transition is associated in most cases with the destruction of the hydrogen bonds between the molecules of the dispersed phase [45,46,47,48] or the specific interactions of the fluoride anions, for example, with silicon-containing ligands (-Si-O bonds) [49,50]; in our case, electrostatic interactions make a major contribution to the process of structuring the dispersed phase molecules and gel formation. Furthermore, electrostatic forces are responsible for the reversibility of the system, that is, they give it a thixotropic nature.
(2) At a certain particle charge value, the system starts losing its colloidal stability, the isotropic sol turns into a structured anisotropic system, a spatial carcass is formed and finally a gel forms.

Due to the structuring of the system, the entropy decreases, and the particles of the dispersed phase form elongated fibers, making more contact with the molecules of the dispersion medium and causing a gain in the enthalpy of the system. This behavior is manifested by all of the LMWGs. Sulfate anions cause such self-assembly [35]. However, in the case of F^−^, abnormal behavior is observed during the formation of a gel network. In accordance with the Hofmeister theory [42,51], the kosmotropic properties of F^−^ and SO_4_^2−^ anions are almost the same, that is, they structure solvent molecules similarly and well. However, fluoride ions, as is well known, form the strongest hydrogen bonds with water molecules compared to other anions [52,53] due to their smallest atomic radius and highest linear charge density [54,55]. Thus, F^−^ leads to the process of twisting the elongated fibers of a gel network into more compact spherical particles owing to the fact that water molecules tend to form the most bonds with the surface of the CSS aggregates. The interactions of the CSS nanoparticles with each other proceed via the electrostatic attractions between protonated amino groups and fluoride anions, as well as NH_3_^+^ and COO^−^. Indeed, small anions like fluoride, but not heavier halides, exhibit a strong affinity to positively charged groups; in contrast, large soft anions such as iodide are weakly attracted to the nonpolar regions of the amino acids [56,57]. Thus, we have obtained the primary information about the self-assembly process in the system under study and suggested the mechanism of gelation, which should be clarified in future experiments.

### 2.4. Toxicity of the Gels to SiHa Cells

**Figure 6:**
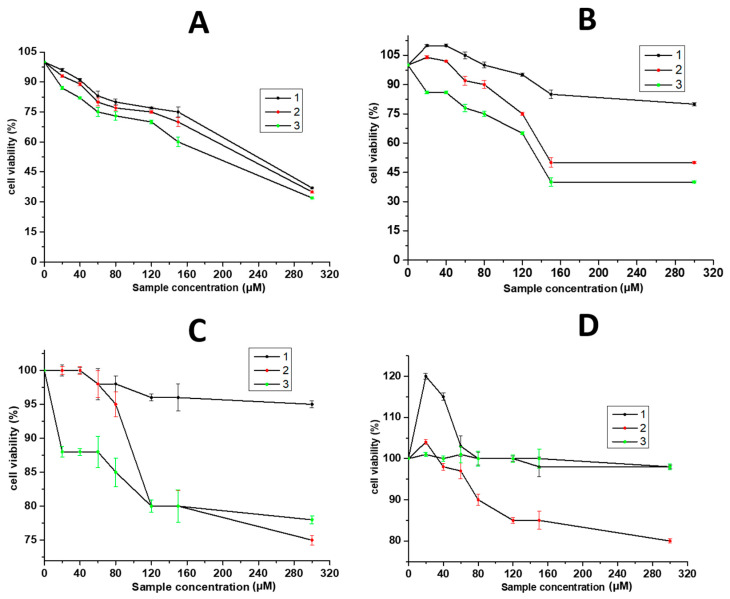
The cytotoxicity (MTT) of F^−^-based CSG against (**A**) SiHa and (**B**) Wi-38 cells at different anion concentrations (mM): **1**—3.1, **2**—3.5, **3**—3.9. The cytotoxicity (MTT) of SO_4_^2−^-based CSG (**1**), CSS (**2**) and NaF (**3**) against (**C**) SiHa and (**D**) Wi-38 cells. SiHa and Wi-38 cell incubation with systems took place for 48 h.

According to the World Health Organization (WHO) statistics, cancer remains the second most common cause of death worldwide [58]. The possibilities of chemotherapy have become more and more limited due to the development of drug resistance, its toxicity, various side effects, etc. Therefore, the synthesis and study of new, safe formulations with high activity is the priority. Stimuli-responsive gels occupy a special place among such compounds [59]. Cancer treatment is performed in most cases via systemic and oral administration; at the same time, local administration could be very useful for non-resectable or incompletely surgically removed tumors. In this context, stimuli-responsive gels based on LMWGs could be prospective materials [5]. For instance, gels obtained from L-alanine derivatives [60], hydrazide/benzaldehyde derivatives [61] and small peptide derivatives [62,63] showed potential applications as matrices for the delivery of various anticancer drugs. We have demonstrated that CSS and SO_4_^2−^-based CSGs suppressed MCF-7 human breast cancer cells with low toxicity toward normal Wi-38 fibroblast cells [36,37]. Here, we studied and compared the activity of CSS, SO_4_^2−^-based CSGs and F^−^-based CSGs against SiHa human squamous carcinoma cells (Figure 6), which is the second most common cutaneous malignancy [64]. All of the F^−^-based CSGs highly suppressed the SiHa cells at the sample concentration of 300 µM (Figure 6A), while the activity of CSS was 2 times lower, and the SO_4_^2−^-based CSGs were practically non-toxic (Figure 6C). The toxicity of the gels based on fluoride anions against the Wi-38 cells was quite high at 300 µM but only for the samples with a high F^−^ concentration (Figure 6B, 1,2). The SO_4_^2−^-based CSGs were non-toxic to the Wi-38 cells, and CSS had a low toxicity (Figure 6D). It is interesting that the intrinsic toxicity of F^−^ against SiHa is similar to that of CSS (Figure 6C, 3) and is the same as that of the SO_4_^2−^-based CSGs against Wi-38 (Figure 6D, 3). Comparing the obtained results, one can conclude that the incorporation of fluoride anions into CSS leads to an additive effect on the resulting system. Herewith, the mechanism of the influence of gels on cells is seemingly related to the toxicity of the fluoride anions themselves, for which the CSS nanoparticles act as a carrier (Figure 5). It is well known that fluoride anions are responsible for bone growth and the dental health of humans at a concentration of lower then 200 µM, which corresponds to organisms’ daily requirement, but excess F^−^ can cause human fluorosis [65]. In vitro and in vivo studies have demonstrated F^−^ can partake in various cellular processes: transport/homeostasis, metabolism, oxidative stress, cell respiration and migration, proliferation, gene expression, signaling, apoptosis, exocytosis, endocytosis, recycling [65]. But the main mechanisms of fluoride ion toxicity are related to protein inhibition, organelle disruption, an altered pH and electrolyte imbalance [66,67,68]. Thus, the obtained gels can be applied to the surface of special sterile films and then directly to affected areas of the skin or mucous membranes.

## 3. Conclusions

In conclusion, the process of the sol–gel transition for CSS using halide anions has been explored. The formation of stable hydrogels with thixotropic properties has been observed for the first time only for F^−^, while the other anions led to precipitation. The self-assembling process proceeds at a fluoride anion concentration from 2.6 to 4.2 mM. The viscosity of the gels slowly decreased on increasing the anion concentration. This fact is explained by the transformation of the elongated structure of the gel network into spherical particles with a diameter of 0.1–2 µm. UV analysis has shown the interaction of F^−^ with the hydrosol does not influence the electronic structure of the CSS nanoparticles. The dynamic light scattering data demonstrated that an increase in the fluoride anion concentration caused growth in the particle size and a decrease in the polydispersity index. Herewith, the zeta potential of the particles gradually drops. The gel formation takes place step by step: fluoride anions condense on the surface of the CSS particles, followed by the interaction of the particles with each other via electrostatic forces and the transition of an isotropic sol to an anisotropic gel. The F^−^-based CSGs showed enhanced anticancer activity toward the SiHa cells compared to conventional CSS and the SO_4_^2−^-based CSGs. The toxicity of the gels toward normal embryonic fibroblasts was low only for the sample with the fewest fluoride anions. Future experiments will be related to using additional instrumental techniques and approaches, including computer simulation, to understand the gel network transformations, as well as investigations of the antibacterial activity and toxicity of the gels to other cell lines.

## 4. Materials and Methods

### 4.1. Reagents

L-cysteine (>99%) was received from Acros. Silver nitrate (>99%) was supplied by Lancaster. The sodium salts, F^−^, Cl^−^, Br^−^, I^−^ and SO_4_^2−^ (pure), were purchased from Vekton. All the reagents were used without any additional purification. The samples under investigation were prepared using deionized water.

### 4.2. Cysteine–Silver Sol and Gel Preparation

To prepare 2 mL of the CSS, our standard previous procedure was used [37]: de-ionized water (0.65 mL) was put into an empty vessel. Then, 0.6 mL of L-cysteine (CYS, 0.01 M) and 0.75 mL of silver nitrate (0.01 M) were added successively. [CYS]/[AgNO_3_] = 1/1.25. The resulting opalescent solution of a white/yellow color was stirred at room temperature (25 °C) for 1 min, and the mixture obtained was put into a dark place for 3 h. A greenish/yellow transparent sol (CSS) was obtained. The CSGs were prepared according to the addition of sodium salts (0.01 M) at different amounts to the CSS in accordance with our midterm results [69]. The hydrogels were stored in a dark place at room temperature.

### 4.3. Vibrational Viscosimetry

An SV-10 (A&D, Tokyo, Japan) vibratory viscometer was used for the viscosity measurements of the samples. The sensor plates’ vibration was carried out at a frequency of 30 Hz and a constant amplitude of about 1 mm. A total of 10 mL of the studied systems was prepared in special polycarbonate cups (A&D) for the viscosity measurements. After 24 h of the samples being retained in a dark place, these cups were transferred to the viscometer, and the measurements were recorded. The temperature of the experiment was 25 °C.

### 4.4. Scanning Electron Microscopy and Elemental Analysis

The microstructure and chemical composition of the samples were studied using a raster JEOL 6610 LV electron microscope (JEOL Ltd., Tokyo, Japan) with X-ray system energy-dispersive microanalysis using an Oxford INCA Energy 350 (JEOL Ltd., Tokyo, Japan) system. The micromorphology of the gels was studied using the high-vacuum mode with an accelerating voltage of 15 kV. For the image acquisition, low-energy secondary electron signals, providing topographical contrast, and high-energy back-up scattered (reflected) electrons, which determine the composition and phase contrast, were generated. The elemental chemical composition of the samples was determined via X-ray spectral microanalysis based on registration and analysis of the energy spectra of the characteristic X-ray radiation excited by the electrons passing through the sample. Its qualitative and quantitative elemental composition was determined using an energy-dispersive spectrometer (EDS), which sorts photons by their energy. The preparation of the samples included spraying the samples onto the thin conductive layer of a platinum surface and drying them in a vacuum (10^−4^ Pa). The average platinum coating time was 5 min.

### 4.5. UV Spectroscopy

The UV spectrophotometer Evolution Array (Thermo Scientific, Waltham, MA, USA) was used to record the electronic spectra of the samples. A quartz cell with a 1 mm path length was used.

### 4.6. Dynamic Light Scattering

A Zetasizer Nano ZS (Malvern, Worcestershire, UK) with a He-Ne laser (633 nm) and a power of 4 mW was used to determine the size of the particles formed in the systems under investigation, as well as their zeta potential. To obtain the correct information about the studied gel systems, the samples were transferred into a sol state by shaking them and finally diluted two, four and eight times. All the measurements were performed at 25 °C in the backscattering configuration at an angle of 173°, which provides the highest sensitivity within the device. Zetasizer software v7.11 was used to mathematically process the obtained results. The cross-correlation function (*g*_2_) was measured, showing the correlation of the scattered light intensity values measured after a time interval *τ*:

$$g_{2}\left(\tau \right)=\frac{<I\left(t\right)*I\left(t+\tau \right)>}{<I^{2}>}$$

If the value of *τ* is small compared to the lifetime of the concentration fluctuation, the value of *g*_2_(*τ*) is the maximum; if *τ* is much longer than the lifetime of the fluctuation, *g*_2_(*τ*) = 0, that is, *g*_2_(*τ*) attenuates with an increase in *τ* from the maximum value to zero. The diffusion coefficient D was calculated according to the attenuation time (the time at which the function decreases by e (2.74) times) of the cross-correlation function:

$$g_{2}\left(\tau \right)=1+C[{\int_{D_{min}}^{D_{max}}Z\left(D\right)exp(-q^{2}*D*\tau )dD]}^{2}$$

*Z*(*D*) is the distribution function of the scattering particles according to the diffusion coefficient. This equation was solved using the cumulant method. As a result, the *Z*(*D*) function was obtained. The hydrodynamic radii of the scattering particles were calculated from the diffusion coefficients using the Stokes–Einstein formula: *D* = kT/6πηR, where *D* is the diffusion coefficient, k is the Boltzmann constant, T is the absolute temperature, η is the viscosity of the medium and R is the radius of the scattering particles.

### 4.7. Zeta Potential Measurements

Measurement of the electrophoretic mobility of the particles in the samples was carried out in U-shaped capillary cuvettes. The zeta potential distributions were calculated using the Henry equation: UE = 2ezf(Ka)/3Z, where UE—electrophoretic mobility, z—zeta potential, e—dielectric constant, Z—viscosity and f (Ka)—Henry’s function; f (Ka) = 1.5 for aqueous media.

### 4.8. MTT-Test

The standard human normal embryonic lung fibroblasts Wi-38 (ATCC, Manassas, VA, USA, Lot. CCL-75) and human squamous cervical carcinoma cells SiHa (ATCC, USA, Lot. HTB-35), commercially available, were obtained from the American Type Culture Collection (ATCC). These cells were not additionally modified genetically; any new data about this culture were received, but they were only used as models of normal or cancer cells to assess the cytotoxicity of the investigated gels. The cells were adhered to 96-well plates and cultured for 24 h at 37 °C in an atmosphere of 5% CO_2_ in a DMEM medium with the addition of L-glutamine (2 mM), antibiotics (100 units per mL of penicillin and 100 μg/mL of streptomycin) and 10% FBS (Diaem, Lot. FBS500SA, Moscow, Russia). The cells were then incubated in a serum medium with the tested compounds of various concentrations for 48 h. PBS was added to the culture medium (10 μL/well), containing MTT (5 mg/mL), and the cells were incubated at 37 °C for 4 h. Yellow tetrazolium bromide (MTT) is converted into purple formazan by cellular respiration. The culture medium was removed, and DMSO (100 μL) was added to each well. The plate was shaken for 20 min at 250 shakes/min at room temperature, and optical absorption measurements were carried out in each well at 570 nm using a Multiskan Spectrum microplate reader (Thermo Scientific, Waltham, MA, USA). The MTT test readings were averaged from three independent experiments using three independent determinations. The MTT test readings in the absence of the test compounds were taken as 100% cell viability.

## Data Availability

All the data and materials are available on request from the corresponding author. The data are not publicly available due to ongoing research using some of the data.
